# Microtubules as Major Regulators of Endothelial Function: Implication for Lung Injury

**DOI:** 10.3389/fphys.2021.758313

**Published:** 2021-10-28

**Authors:** Pratap Karki, Anna A. Birukova

**Affiliations:** Division of Pulmonary and Critical Care Medicine, Department of Medicine, University of Maryland School of Medicine, Baltimore, MD, United States

**Keywords:** microtubules, endothelial barrier, cytoskeletal remodeling, inflammation, acute lung injury, histone deacetylase

## Abstract

Endothelial dysfunction has been attributed as one of the major complications in COVID-19 patients, a global pandemic that has already caused over 4 million deaths worldwide. The dysfunction of endothelial barrier is characterized by an increase in endothelial permeability and inflammatory responses, and has even broader implications in the pathogenesis of acute respiratory syndromes such as ARDS, sepsis and chronic illnesses represented by pulmonary arterial hypertension and interstitial lung disease. The structural integrity of endothelial barrier is maintained by cytoskeleton elements, cell-substrate focal adhesion and adhesive cell junctions. Agonist-mediated changes in endothelial permeability are directly associated with reorganization of actomyosin cytoskeleton leading to cell contraction and opening of intercellular gaps or enhancement of cortical actin cytoskeleton associated with strengthening of endothelial barrier. The role of actin cytoskeleton remodeling in endothelial barrier regulation has taken the central stage, but the impact of microtubules in this process remains less explored and under-appreciated. This review will summarize the current knowledge on the crosstalk between microtubules dynamics and actin cytoskeleton remodeling, describe the signaling mechanisms mediating this crosstalk, discuss epigenetic regulation of microtubules stability and its nexus with endothelial barrier maintenance, and overview a role of microtubules in targeted delivery of signaling molecules regulating endothelial permeability and inflammation.

## Introduction

Endothelial barrier formed by a lining of heterogenous mixture of endothelial cells (EC) is a highly selective semi-permeable barrier between the blood and interstitial space that is critical for maintaining tissue and organ homeostasis (Bazzoni and Dejana, 2004; Chistiakov et al., 2015). The disruption of EC barrier in lung endothelium results in influx of fluids, macromolecules, pathogens, and immune cells into the lung air spaces, causing pulmonary edema which is a pathological hallmark of multiple lung diseases such as acute lung injury (ALI), acute respiratory distress syndrome (ARDS), and sepsis (Maniatis et al., 2008; Maniatis and Orfanos, 2008; Opal and van der Poll, 2015). The endothelial barrier dysfunction in other organs have also been implicated in the pathogenesis of various diseases including brain edema, stroke, cognitive impairment, atherosclerosis, metabolic disorders, and cancers (Rodrigues and Granger, 2015; Park-Windhol and D’Amore, 2016; Profaci et al., 2020). More importantly, a number of studies have reported that endothelial barrier dysfunction is a major pathological feature of current global pandemic coronavirus disease 2019 (COVID-19) (Chang et al., 2020; Gavriilaki et al., 2020; Huertas et al., 2020; Jin et al., 2020; Sims et al., 2021). The endothelial barrier integrity is maintained by cell junction adhesive protein complexes linked to actin cytoskeleton, and increasing evidence suggests that microtubules (MT) are integral component of this interactive unit (Sukriti et al., 2014; Kasa et al., 2015). The breach of endothelial barrier is caused by the breakdown of adherens junction (AJ) and/or tight junction (TJ) protein complexes *via* site-specific protein phosphorylation events leading to the paracellular gap formation. Another mechanism of EC permeability involves Rho GTPase-mediated remodeling of actin cytoskeleton and an increase in actomyosin contractility leading to cell retraction and uncoupling of homotypic interactions between transmembrane adhesive proteins (i.e., cadherins, occludins, claudins, nexins) (Wojciak-Stothard and Ridley, 2002; Bazzoni and Dejana, 2004; Wallez and Huber, 2008; Spindler et al., 2010; Komarova et al., 2017). Recent studies have uncovered an equally important role of MT dynamics in regulating endothelial barrier function (Lee and Gotlieb, 2003; Birukova et al., 2004c; Alieva et al., 2013).

Microtubules are dynamic tubular intracellular structures composed of α-, β-tubulin heterodimers and MT-associated regulatory proteins. MT mediate important cellular functions such as cell division, motility, cell shape changes, intracellular transport, and cellular organization (Nogales, 2000; Goodson and Jonasson, 2018). In addition to these conventional functions of MT, changes in MT dynamics caused by vasoactive or inflammatory agonists and mechanical forces play essential role in control of EC permeability *via* signaling crosstalk with EC junctions and actin cytoskeleton. This review will discuss presently known mechanisms of endothelial barrier regulation by MT signaling nexus.

### Microtubules in Control of Endothelial Permeability: Role and Mechanisms

**FIGURE 1:**
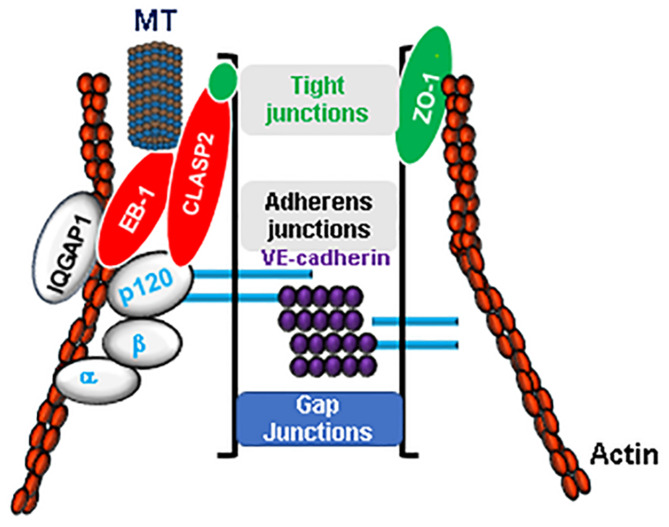
Microtubules (MT) are integral component of endothelial barrier. The integrity of endothelial barrier is maintained by a complex interaction between adherens junctions (VE-cadherin, α, β, and p120 catenins), tight junctions (ZO-1, Claudins, Occludin) proteins and actin cytoskeleton. MT are in firm association with EC junctions by their close interaction *via* MT plus-end-tracking proteins such as CLASP2 and EB-1. Furthermore, cytoskeletal scaffold protein IQGAP1 serves as a linker for an active crosstalk between MT and actin cytoskeleton.

Cytoskeleton remodeling driven by a balance between contractile and tethering forces determines the endothelial barrier integrity under both physiological and pathological states (Kasa et al., 2015). Endothelial barrier is formed and maintained by intercellular junctions represented by TJ and AJ that are composed of multiprotein complexes (Figure 1). A dynamic interaction of these proteins facilitates their association with actin cytoskeleton and maintains the integrity of endothelial barrier. More recent studies have established an essential role of MT in regulating endothelial barrier function since a number of MT-associated proteins are also involved in dual interaction with EC junction proteins and actin cytoskeleton. As an integral components of cytoskeleton, MT dynamics directly controls endothelial permeability by altering cytoskeletal organization *via* regulation of actomyosin contractility, paracellular gaps and stress fibers formation (Lee and Gotlieb, 2003; Smurova et al., 2008). More importantly, alteration of MT dynamics appears to be the first target during agonist-induced endothelial barrier disruption. This notion was supported by the findings that thrombin treatment caused peripheral MT disassembly as early as 5 min but required 30 min for formation of actin stress fibers (Alieva et al., 2013). These results suggest that changes in MT structure precede microfilaments reorganization during barrier disruptive agents-induced increase in endothelial permeability. Studies have now established that a pool of stable MT favors endothelial barrier integrity, while disassembly of MT network has been associated with an increase in endothelial permeability. In this regard, inhibitors of MT polymerization such as nocodazole directly cause an increase in endothelial permeability which can be prevented by cell pretreatment with MT stabilizer paclitaxel (Verin et al., 2001). In addition to these plant-derived MT poisons, MT growth and MT stability can be also regulated by endogenous circulating bioactive molecules. For example, a rapid and reversible partial dissolution of peripheral MT has been reported in pulmonary EC stimulated with thrombin and is associated with rapid permeability increase (Birukova et al., 2004a). In support of this MT-dependent mechanism, cell pretreatment with MT stabilizing agent taxol rescued thrombin-induced increase in endothelial permeability by attenuating Rho activation, suggesting an essential role of MT dynamics in controlling endothelial barrier function during agonists challenges. Later on, numerous *in vitro* and *in vivo* models of ALI studies, including our own, have provided substantial evidence that MT destabilization is a critical step during endothelial barrier disruption.

Investigations into mechanism of MT-dependent modulation of agonist-induced EC permeability showed that partial depolymerization of peripheral MT promoted, while stabilization prevented activation of the RhoA signaling pathway of endothelial permeability. RhoA GTPase serves as a molecular switch by cycling between GTP-bound active and GDP-bound inactive states and thus regulates various cell functions including actin cytoskeletal reorganization (Siderovski and Willard, 2005; Beckers et al., 2010; Karki and Birukov, 2019). The switch between active and inactive forms of Rho GTPase is regulated by various proteins. Guanine nucleotide exchange factors (GEFs) promote activated, GTP-bound state of RhoA while GDP dissociation inhibitors (GDIs) and GTPase activating proteins (GAPs) inhibit RhoA activity (Siderovski and Willard, 2005). Activated Rho, through its effector Rho kinase, activates actomyosin contractility and EC permeability either directly by phosphorylating myosin light chain (MLC) or indirectly, *via* inhibition of myosin phosphatase (MYPT1) leading to increased phospho-MLC levels (Garcia et al., 1995; Amano et al., 1996; Kimura et al., 1996). A direct link between Rho activation, MT disassembly and endothelial hyperpermeability was established by the observation that ectopic expression of activated Rho or Rho kinase induced MT disassembly, while overexpression of dominant negative RhoA and Rho-kinase mutants attenuated thrombin-induced peripheral MT dissolution (Birukova et al., 2004a). Consistent with the proposed MT-RhoA-actin cytoskeleton crosstalk mechanism, MT-dependent endothelial barrier disruption was mediated by Rho-mediated MLC phosphorylation, cytoskeletal remodeling, formation of actin stress fibers and paracellular gaps (Verin et al., 2001; Birukova et al., 2004a,c). Further studies revealed that thrombin-induced peripheral MT depolymerization leading to RhoA activation and endothelial permeability was associated with increased MT instability caused by thrombin-induced phosphorylation of MT regulatory protein tau at Ser^409^ and Ser^262^ (Birukova et al., 2004a). A similar mechanism of endothelial hyperpermeability was described in pulmonary EC challenged with transforming growth factor-beta1 (TGF-β1). A partial dissolution of peripheral MT network has also been associated with a decreased pool of acetylated stable MT (Birukova et al., 2005a). Inhibition of the RhoA pathway, MT stabilization or forskolin-mediated elevation of intracellular cAMP attenuated TGF-β1-induced MT disassembly and endothelial barrier disruption. Another study demonstrated the protective effects of heat shock protein HSP90 pharmacologic inhibitors against endothelial permeability caused by various agonists including TGF-β1 which involved the restoration of peripheral MT organization, inhibition of MLC/MYPT1 phosphorylation and actin stress fibers formation (Antonov et al., 2008).

RhoA-independent mechanism of MT disassembly and subsequent endothelial barrier disruption has also been described. The decrease in a stable pool of MT and a partial dissolution of peripheral MT network during EC permeability caused by tumor necrosis factor-α (TNF-α) was linked to the activation of p38 mitogen-activated kinase and subsequent destabilization of MT (Petrache et al., 2003). In agreement with these results, inhibition of p38 MAPK directly suppressed nocodazole-induced MT depolymerization and endothelial barrier disruption (Birukova et al., 2005b). Moreover, activation of p38 MAPK mediated 2-Methoxyestradiol-induced MT depolymerization and endothelial barrier disruption (Bogatcheva et al., 2007). A recent report showed that p38 MAPK-dependent phosphorylation of MT-associated protein 4 (MAP4) caused MT disruption, and this mechanism was involved in lipopolysaccharide (LPS) or TNF-α-induced endothelial permeability (Li et al., 2015). In contrast to RhoA and p38-kinase, activation of protein kinase A (PKA) stabilizes MT network and attenuates nocodazole-induced endothelial barrier dysfunction *via* suppression of Rho activation, MLC phosphorylation, and stress fiber formation (Birukova et al., 2004b). Similarly, protein phosphatase 2A (PP2A) was found to be associated with MT in endothelium, and inhibition of PP2A caused disassembly of MT *via* tau phosphorylation leading to an exacerbation of nocodazole-induced endothelial barrier disruption (Tar et al., 2004). Consistently, overexpression of PP2A in ECs protected against thrombin or nocodazole-induced endothelial barrier disruption *via* inhibition of tau phosphorylation and MT dissolution (Tar et al., 2006). Exotoxin Y from *Pseudomonas aeruginosa* is known to increase endothelial permeability (Ochoa et al., 2012). Interestingly, this toxin directly caused MT disassembly and impaired MT reassembly *via* tau phosphorylation (Balczon et al., 2013).

**FIGURE 2:**
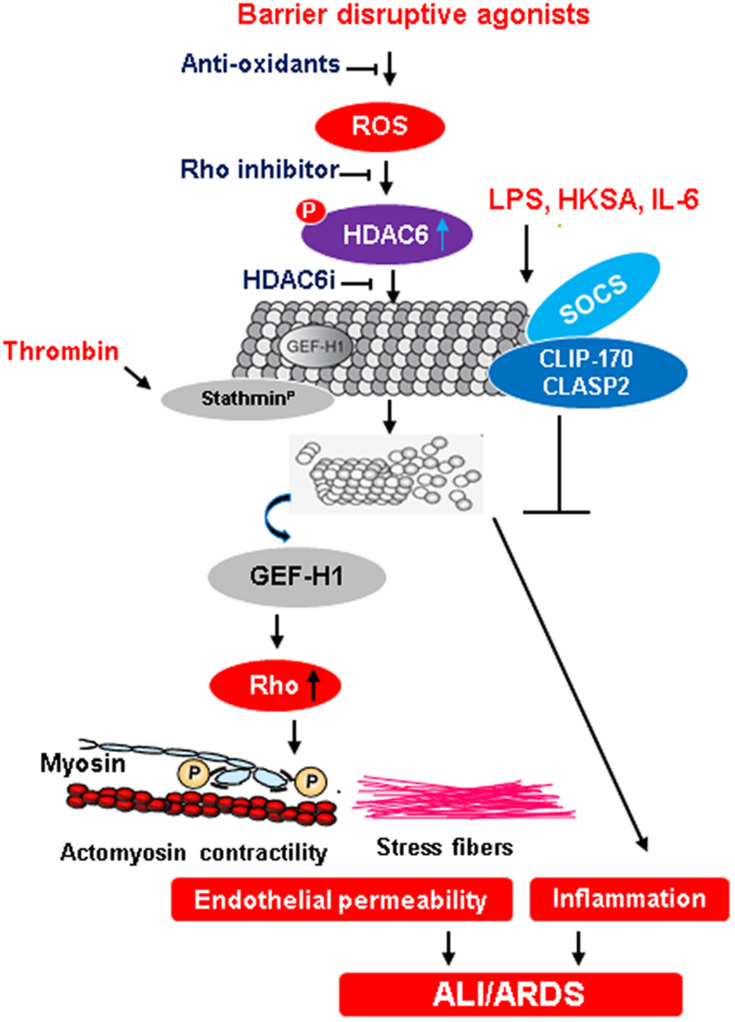
MT-mediated regulation of endothelial barrier function during ALI/ARDS. An excessive ROS production in response to injurious stimuli activates HDAC6 by phosphorylation. In turn, activated HDAC6 deacetylates tubulin causing the destabilization of MT and release of Rho-specific GEF-H1. The activation of Rho signaling pathway induces cytoskeletal remodeling with increased actomyosin contractility and stress fibers formation that leads to an increase in endothelial permeability, a trigger for ALI and ARDS. Anti-oxidants and inhibition of Rho as well as HDAC6 presents points of potential therapeutic interventions to prevent endothelial dysfunction-derived lung injuries. Dephosphorylation of stathmin by thrombin also contributes to MT destabilization and subsequent endothelial dysfunction. Conversely, the formation of a protein complex between SOCS and MT plus-end-tracking proteins CLIP-170 and CLASP2 exerts anti-inflammatory effects on EC.

Oxidative stress-induced alteration of MT dynamics appears to be another common mechanism of endothelial dysfunction caused by bacterial pathogens and inflammatory agonists (Figure 2). For example, LPS-induced increase in reactive oxygen species (ROS) production caused the disassembly of MT, activation of Rho, p38MAPK, and NF-κB signaling pathways leading to endothelial hyperpermeability and inflammation (Kratzer et al., 2012). Likewise, MT destabilization caused by elevated ROS levels has been implicated in the endothelial dysfunction caused by heat-killed *Staphylococcus aureus* (HKSA) and air pollution particulate matter (Karki et al., 2019a,b). At this point, it is not clear which intermediate molecules that trigger MT destabilization are activated in response to elevated ROS levels. Further studies are also required to explore whether inhibition of ROS production is sufficient for restoration of MT destabilization-induced endothelial dysfunction and if additional mechanisms such as disruption in interaction of MT-associated proteins with other components of EC barrier are involved.

### GEF-H1: A Key Player in Microtubules-Mediated Endothelial Dysfunction

GEF-H1, a Rho-specific GEF, has been identified as a key signaling molecule of an active cross-talk between MT and actin cytoskeleton (Ren et al., 1998; Krendel et al., 2002). GEF-H1 bound to MT remains in an inactive state, but its release from MT stimulates GEF-H1 nucleotide exchange activity. In consistence with MT-dependent mechanism of GEF-H1 activity regulation, GEF-H1 mutants lacking MT binding sites exhibit increased activity and induce Rho-dependent actin cytoskeleton reorganization (Krendel et al., 2002). A direct role of GEF-H1 in agonist-induced, Rho-dependent and MT disassembly-driven endothelial dysfunction has been clearly elucidated with the findings that knockdown of GEF-H1 attenuated thrombin- or nocodazole-induced EC barrier disruption, while overexpression of wild type GEF-H1 exacerbated such barrier disruptive effects (Birukova et al., 2006). This notion was further validated in cultured human pulmonary EC subjected to high magnitude cyclic stretch (18% CS) relevant to ventilator-induced lung injury (VILI). Exposure to 18% CS induced MT disassembly and Rho-dependent endothelial dysfunction in pulmonary EC, which was attenuated by knockdown of GEF-H1 (Birukova et al., 2010). Protective effects of atrial natriuretic peptide (ANP) against thrombin-induced MT disassembly and Rho-mediated endothelial dysfunction were also mediated by inhibitory phosphorylation of GEF-H1 by Rac1 GTPase effector, PAK1 kinase (Tian et al., 2014a). Thus, a growing body of evidence suggests that activation of GEF-H1 acts as a common mechanism exacerbating pulmonary EC inflammation and barrier dysfunction caused by pathologic mechanical (high magnitude cyclic stretch, increased substrate stiffness) and chemical (LPS, cytokines) stimulation (Mambetsariev et al., 2014; Yi et al., 2017). In addition to MT-associated Rho activator GEF-H1, Rho inhibitor GAP ARHGAP18 localizes on MT and its depletion causes MT destabilization (Lovelace et al., 2017). It was demonstrated that binding of GEF-H1 with TJ protein cingulin rescues thrombin-induced endothelial permeability by inactivating the Rho pathway (Tian et al., 2016). It will be interesting to investigate whether MT dynamics play an essential role in maintaining such interactions to regulate endothelial function.

### Microtubules-Associated Rac Signaling in Enhancement of Endothelial Barrier Function

**FIGURE 3:**
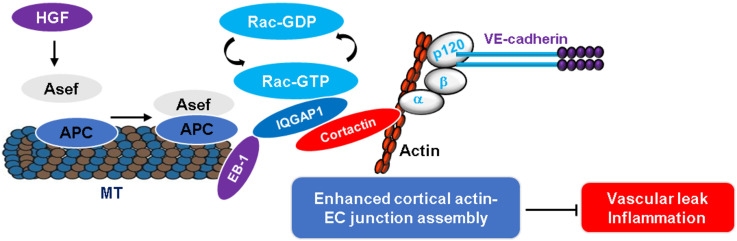
MT-associated Rac regulation of endothelial barrier function. HGF stimulation of EC induces Asef, a Rac-specific GEF, activation which undergoes peripheral translocation by forming a complex with MT protein APC. In turn, Asef associates with IQGAP1 at the cell periphery and activates Rac, resulting in enhanced endothelial barrier integrity. The upregulated endothelial barrier function inhibits agonists-induced vascular leak and inflammation.

Basal Rac1 GTPase activity is essential for maintaining endothelial barrier function in resting cells, and its additional activation mediates EC barrier enhancement induced by various barrier protective agents (Mehta and Malik, 2006; Birukova et al., 2007). In this regard, the role of MT-dependent Rac signaling in the regulation of endothelial barrier integrity has been investigated by several groups. The findings from these studies uncovered a crucial role of Asef, a Rac-specific GEF, in MT-dependent endothelial barrier enhancement induced by hepatocyte growth factor (HGF) (Figure 3; Higginbotham et al., 2014; Tian et al., 2014b; Tian X. et al., 2015). Asef has been implicated in cytoskeleton remodeling (Kawasaki et al., 2000, 2010) and it is composed of a Dbl homology domain with GEF activity, plekstrin homology domain determining the subcellular localization, Src homology 3 autoinhibitory domain, and a binding region for tumor suppressor adenomatous polyposis coli protein (APC) (Kawasaki et al., 2003; Gotthardt and Ahmadian, 2007; Murayama et al., 2007). HGF stimulates Rac1-specific activity of Asef by inducing its membrane translocation where it forms a complex with MT-associated protein APC (Tian X. et al., 2015). Moreover, the ectopic expression of constitutively active form of Asef mimics cytoskeletal remodeling induced by HGF and depletion of endogenous Asef or overexpression of dominant negative Asef abolishes the barrier protective effects of HGF, suggesting an essential role of Asef in HGF-induced positive regulation of endothelial barrier (Tian X. et al., 2015). An association of Asef with growing MT tips is essential for HGF-induced endothelial barrier enhancement, as Asef-APC complex is increased in MT enriched fractions upon HGF stimulation (Higginbotham et al., 2014). In turn, inhibition of MT peripheral growth suppresses Asef translocation to cell periphery, resulting in attenuation of Rac1 activation and subsequent reduction in endothelial barrier enhancement. A critical role of Asef in the regulation of endothelial function was further validated in *in vivo* studies where HGF-induced protection against LPS- or thrombin-induced lung injury was abolished in Asef knockout mice (Meng et al., 2015; Tian X. et al., 2015).

### Microtubules-Associated Proteins in Modulating Endothelial Function

A number of proteins act as linkers between MT and actin cytoskeleton, thereby regulating MT-mediated cytoskeletal remodeling in response to altered MT dynamics. Among these, a multifunctional adaptor protein IQGAP1 is known to control MT as well as cytoskeletal dynamics by its interaction with Rac as its effector molecule (Swart-Mataraza et al., 2002; Noritake et al., 2005). Furthermore, IQGAP1 also directly interacts with APC and MT plus-end-tracking protein CLIP-170 (Fukata et al., 2002; Watanabe et al., 2004). Interestingly, HGF-induced enhancement of EC barrier function was dependent upon the formation of Asef-Rac-IQGAP1 protein complex at the cell cortical area (Tian Y. et al., 2015). This interaction was facilitated by an interaction between C-terminus of IQGAP1 and active Asef with nucleotide exchange activity. Another MT-associated protein end-binding protein 1 (EB1) also forms a protein complex with IQGAP1 and cortactin following HGF stimulation to mediate the barrier enhancing effects of HGF (Tian et al., 2014c). HGF-stimulated EC showed EB1 distribution to the cell periphery, while EB1 knockdown suppressed Rac1 activation and attenuated HGF-induced enhancement of cortical actin and AJ assembly (Tian et al., 2014c). These findings further strengthened the role of EB1 in coordination of MT-dependent EC barrier function. Stathmin, another MT-associated protein, also controls MT dynamics and agonist-induced endothelial dysfunction. Thrombin-induced dephosphorylation of stathmin disrupts MT network and escalates RhoA-mediated endothelial barrier dysfunction (Tian et al., 2012). Accordingly, knockdown of stathmin attenuated thrombin-induced endothelial permeability *in vitro* and also suppressed lung vascular leak in the 2-hit mouse model of ALI caused by mechanical ventilation combined with injection of thrombin receptor activating peptide (Tian et al., 2012). The phosphorylation-mediated inactivation of stathmin contributed to ANP-induced restoration of endothelial permeability caused by *Staphylococcus aureus-derived* peptidoglycan G (Tian Y. I et al., 2015) and was associated with MT destabilization. In that study, ANP suppressed peptidoglycan G-induced MT disassembly, GEF-H1-Rho activation, and inflammatory NF-κB signaling which were abolished upon transient expression of phosphorylation-deficient stathmin mutant. ANP-mediated protection of peptidoglycan G-induced lung injury in mice was associated with increased levels of phosphorylated stathmin, while mice with ANP knockout showed exacerbated lung injury with decreased pools of stable MT and this effect was reversed by stathmin knockdown (Tian Y. I et al., 2015). MAP4 is a cytosolic MT-binding protein that upon phosphorylation disassociates from tubulin and causes MT instability (Ebneth et al., 1999; Hu et al., 2014). As already mentioned above, p38MAPK-mediated serine phosphorylation of MAP4in response to LPS and TNF-α stimulation causes MT disruption leading to endothelial permeability (Li et al., 2015).

### Microtubules in Endothelial Inflammation

Excessive inflammatory response is another major feature of endothelial dysfunction that contributes to severe lung injury (Maniatis et al., 2008). Increased endothelial permeability and aggravated inflammation are coordinated responses triggered by pathologic factors and occur in parallel. As such, altered MT dynamics also has been linked to inflammation, and MT stabilizer taxol has been proven to be effective in protecting against both, LPS-induced vascular leak and inflammation *in vivo* (Mirzapoiazova et al., 2007). Taxol also improved the function of isolated rat lungs during cold preservation that might have significant clinical impacts for lung transplantations (Suzuki et al., 2004). Of note, taxol has been commonly employed as a chemotherapeutic drug against breast, ovarian and lung cancers and its use has been often associated with various side effects including hypersensitivity reactions, neuropathy, myelotoxicity and lung injuries (Raghupathi et al., 2021). In this regard, a study has reported that paclitaxel treated rats show increased inflammatory responses in the lung tissues and an increased vascular permeability with decreased expression of TJ proteins ZO-1 and claudin-4 (Liu et al., 2015). Thus further studies are required to evaluate the beneficial effects of taxol in preventing vascular leak and inflammation during lung injuries.

The increased expression of EC adhesion molecules intercellular adhesion molecule-1(ICAM-1) and vascular cell adhesion molecule-1 (VCAM-1) is a prerequisite for transendothelial migration of immune cells into affected organs and induce inflammation. It has been reported that ANP-mediated MT stabilization attenuated Peptidoglycan G-induced expression of ICAM-1and VCAM-1 (Tian Y. I et al., 2015). Likewise, other agonists such as HKSA and particulate matter that induce endothelial dysfunction *via* MT depolymerization are shown to activate inflammatory NF-κB signaling pathway and upregulate the expression of ICAM-1 and VCAM-1 (Karki et al., 2019a,b). A family of suppressor of cytokine signaling (SOCS) proteins also inhibit LPS- and HKSA-induced activation of the NF-κB pathway and expression of ICAM-1 and VCAM-1 in MT-dependent manner (Karki et al., 2021a,b). These mechanisms will be discussed in the later section. As in the case of permeability, endothelial inflammation may be also mediated by the activation of RhoA pathway, as shown for expression of ICAM-1 induced by thrombin and lysophosphatidic acid (Anwar et al., 2004; Shimada and Rajagopalan, 2010). Nonetheless, the precise role of altered MT dynamics in inducing inflammation is not as clear as in the case of endothelial permeability and future studies are warranted to illustrate the mechanisms of endothelial inflammation promoted by MT disassembly.

### Histone Deacetylase 6: Microtubules-Associated Deacetylase as a Therapeutic Target in Lung Injury Syndromes

Post-translational modifications of tubulin regulates the structure and function of MT (Janke and Bulinski, 2011). In particular, acetylation of tubulin at Lys^40^ confers MT stability (Portran et al., 2017; Xu et al., 2017), whereas HDAC6-mediated deacetylation is linked to destabilization of MT (Hubbert et al., 2002; Matsuyama et al., 2002; Zilberman et al., 2009). HDAC6 is a class IIb HDAC that is exclusively localized in the cytoplasm and directly associates with MT, α-tubulin being its preferred substrate (Hubbert et al., 2002; Matsuyama et al., 2002). As therapeutic potential of HDAC6 inhibition has been evaluated in cardiovascular, neurodegenerative diseases and cancers, recent studies have assessed the role of HDAC6 inhibition in endothelial dysfunction-derived ALI (Kovacs et al., 2018; Zhang et al., 2021). Initial studies showed the protective effects of HDAC6 inhibitors against thrombin-induced permeability in EC culture (Saito et al., 2011) and attenuation of LPS-induced ALI in mice (Ni et al., 2010). Likewise, HDAC6 inhibition by its pharmacological inhibitor tubastatin A or siRNA-mediated gene knockdown exerted protection against LPS-induced endothelial barrier disruption and ALI (Joshi et al., 2015). Concurrently, other studies have demonstrated the protective effects of HDA6 inhibition *via* MT stabilization against other barrier disruptive agents including TNF-α (Yu et al., 2016b) and cigarette smoke extract (Borgas, 2016). These findings are further supported by recent studies where HDAC6 activation-driven MT disruption was a key in mediating endothelial dysfunction caused by *Staphylococcus aureus* and air pollution particulate matter (Karki et al., 2019a,b). Notably, all aforementioned studies suggest that HDAC6-mediated tubulin deacetylation resulting in MT depolymerization is the major cause of endothelial dysfunction. Briefly, HDAC6-mediated MT destabilization either directly causes the disassembly of AJs or releases GEF-H1 to activate Rho signaling pathway, both ultimately resulting in increased endothelial permeability and inflammation (Borgas, 2016; Yu et al., 2016b; Karki et al., 2019a,b). Elevated ROS production or activated glycogen synthase kinase (GSK)-3β-mediated serine phosphorylation of HDAC6 appears to act as the upstream activator of HDAC6 (Borgas, 2016; Karki et al., 2019a). Also, a recent study has shown that LPS challenge stimulates HDAC6 activity and HDAC6 inhibitor CAY10603 rescues LPS-induced ALI by restoring tubulin acetylation, and inhibition of cytokine storm and inflammasome (Liu et al., 2019). Nevertheless, a few studies have suggested additional mechanisms of ALI rescue by HDAC6 inhibitors *via* anti-apoptotic and anti-inflammatory activities, reassembly of EC junctions, and inhibition of RhoA signaling (Joshi et al., 2015; Yu et al., 2016a; Liu et al., 2019). A recent study has shown that inhibitors of other HDACs classes also protect against endothelial barrier dysfunction and ALI, but these processes are independent of MT dynamics (Kovacs-Kasa et al., 2021). It indicates that HDAC-mediated epigenetic programming also involves other beneficial mechanisms to combat against endothelial dysfunction beyond HDAC6-mediated control of MT dynamics that needs future attention.

As discussed above, restoration of endothelial functions by barrier-protective agonists involves MT stabilization. In this regard, a recent study has shown that attenuation of LPS-induced endothelial dysfunction caused by unfractionated heparin was associated with restoration of tubulin acetylation levels (Mu et al., 2018). Another study has reported that dopamine D1 receptor (DRD1)-mediated protection of endothelial barrier dysfunction caused by CS involves the inactivation of HDAC6 and maintenance of MT stability (Wang et al., 2021). HDAC6 inhibitors have been also found to be effective against other lung diseases such as pulmonary fibrosis (Saito et al., 2017), and pulmonary arterial hypertension (Boucherat et al., 2017), the conditions associated with altered mechano-chemical microenvironment of lung endothelium. Of note, MT stabilization-mediated protection of endothelial barrier integrity is not restricted to lung endothelium as exemplified by taxol-induced protection of corneal EC barrier (Jalimarada et al., 2009; Shivanna and Srinivas, 2009).

Activation of Rho GTPases and HDAC6 appear to act in coordinated manner to cause MT-driven endothelial dysfunction. Activation of RhoA causes MT destabilization *via* stimulation of HDAC6 activity and conversely HDAC6-driven MT disassembly leads to increased Rho activity, ultimately causing cytoskeletal reorganization associated with endothelial barrier disruption (Schofield et al., 2012). Some proteins are also involved in cross-talk between Rho GTPases and HDAC6 to control MT dynamics. For instance, tubulin polymerization promoting protein 1 (TPP1) favors MT stability by inhibiting HDAC6 activity (Schofield et al., 2013). In turn, Rho-associated coiled-coil kinase (ROCK)- mediated phosphorylation of TPP1 abolishes its HDAC6 inhibition activity and inhibits MT polymerization (Schofield et al., 2013).

### Suppressor of Cytokine Signaling-Microtubules Interaction: A Novel Microtubules-Mediated Regulatory Mechanism of Endothelial Function

SOCS represent a family of cytokine-inducible intracellular proteins that act as key regulators of cytokine signaling involved in immunity and inflammation (Alexander, 2002; Yoshimura et al., 2007). The latest studies have unraveled unique mechanisms of SOCS-mediated regulation of endothelial function in MT-dependent fashion (Karki et al., 2021a,b). These studies explored the barrier protective and anti-inflammatory modalities of two closely related SOCS members: SOCS1 and SOCS3 on lung endothelium. The results demonstrated that both SOCS1 and SOCS3 protected against inflammatory agonist-induced endothelial permeability and inflammation in MT-dependent manner. SOCS were enriched in MT fractions, while stimulation with inflammatory agonists disrupted SOCS-MT association, and even partial MT disassembly with nocodazole abolished beneficial effects of SOCS on lung EC (Karki et al., 2021a,b). A comprehensive mechanistic analysis showed that SOCS1 and SOCS3, *via* their N-terminal domains, directly interact with MT plus-end-binding proteins CLIP-170 and CLASP2. More importantly, MT depolymerization not only inhibits the MT-SOCS interaction but also abolishes the protective actions of SOCS on lung endothelium. Multifunctional cytoskeletal scaffold protein IQGAP1 facilitates the delivery, anchoring and stability of anti-inflammatory SOCS-MT signaling complex at the submembrane regions. These findings suggest that proper targeting to MT and interaction with MT-associated proteins is essential for beneficial effects of SOCS on lung endothelium. It indicates that in addition to altered MT dynamics, the interaction of MT proteins with barrier protective molecules is also equally crucial for MT-mediated regulation of endothelial function. It not only establishes an unprecedented role of MT in regulating anti-inflammatory signaling hub but also warrants further investigation into its mechanistic details and existence of such interaction between MT proteins and inflammation resolving targets under real clinical scenario.

### Summary

An active and intimate cross-talk between MT and actin cytoskeleton is essential for maintaining endothelial barrier integrity. The altered MT dynamics, specifically depolymerization of MT, is a crucial trigger for endothelial dysfunction and studies have now established that MT destabilization contributes to various barrier disrupting agonists-induced ALI. Accordingly, MT stabilizing drugs and epigenetic modifiers such as HDAC6 inhibitors that favor MT polymerization have shown promising therapeutic potential in a wide range of *in vitro* and *in vivo* models of lung injuries. Mechanistically, activation of the Rho signaling pathway evoked by GEF-H1 release from depolymerized MT appears to be the major driving force for MT-derived endothelial dysfunction. On the other hand, MT-dependent Rac activation is crucial for agonist-induced upregulation of endothelial barrier function. Additionally, MT-associated proteins such as stathmin, MAP4 and direct interaction of MT-plus-end tracking proteins CLIP-170, CLASP2 with endothelial junction proteins also regulate endothelial barrier function. The latest findings that anti-inflammatory proteins SOCS protect endothelial function *via* their interaction with MT opens a new avenue to pursue such novel MT-dependent mechanisms of EC regulation. In summary, exploring the mechanisms that preserve stable pools of MT and maintain their dynamic interaction with actin to protect endothelial barrier function presents an attractive therapeutic approach to combat against endothelial dysfunction-derived lung injuries such as ALI which still holds an alarming situation with 200,000 cases annually and 30–40% mortality in the United States.

## Author Contributions

PK and AB designed the review outline. PK drafted the manuscript. AB edited the manuscript. Both authors contributed to the article and approved the submitted version.

## Conflict of Interest

The authors declare that the research was conducted in the absence of any commercial or financial relationships that could be construed as a potential conflict of interest.

## Publisher’s Note

All claims expressed in this article are solely those of the authors and do not necessarily represent those of their affiliated organizations, or those of the publisher, the editors and the reviewers. Any product that may be evaluated in this article, or claim that may be made by its manufacturer, is not guaranteed or endorsed by the publisher.

## Abbreviations

MT: microtubules
EC: endothelial cells
ALI: acute lung injury
ARDS: acute respiratory distress syndrome
AJ: adherens junction
TJ: tight junction
MLC: myosin light chain
MYPT1: myosin phosphatase 1
GEF: guanine nucleotide exchange factor
TNF-α: tumor necrosis factor-α
TGF-β: transforming growth factor-β
LPS: lipopolysaccharide
PP2A: protein phosphatase 2 A
PKA: protein kinase A
MAP4: microtubule-associated protein 4
ANP: atrial natriuretic peptide
HGF: hepatocyte growth factor
CS: cyclic stretch
VILI: ventilator-induced lung injury
APC: adenomatous polyposis coli protein
VCAM-1: vascular cell adhesion molecule-1
ICAM-1: intercellular adhesion molecule-1
HDAC6: histone deacetylase 6
SOCS: suppressor of cytokine signaling.
